# Integration of stress and leptin signaling by CART producing neurons in the rodent midbrain centrally projecting Edinger-Westphal nucleus

**DOI:** 10.3389/fnana.2014.00008

**Published:** 2014-03-03

**Authors:** Lu Xu, Donny Janssen, Noortje van der Knaap, Eric W. Roubos, Rebecca L. Leshan, Martin G. Myers, Balázs Gaszner, Tamás Kozicz

**Affiliations:** ^1^Department of Anatomy, Donders Institute for Brain, Cognition and Behaviour, Radboud University Nijmegen Medical CentreNijmegen, Netherlands; ^2^Department of Cognitive Neuroscience, Donders Institute for Brain, Cognition and Behaviour, Radboud University Nijmegen Medical CentreNijmegen, Netherlands; ^3^Division of Metabolism, Endocrinology and Diabetes – Department of Internal Medicine, University of Michigan, Ann ArborMI, USA; ^4^Department of Anatomy, University of PécsPécs, Hungary

**Keywords:** *db/db* mice, depression, centrally projecting Edinger–Westphal nucleus, fasting, obesity, restraint

## Abstract

Leptin targets the brain to regulate feeding, neuroendocrine function and metabolism. The leptin receptor is present in hypothalamic centers controlling energy metabolism as well as in the centrally projecting Edinger–Westphal nucleus (EWcp), a region implicated in the stress response and in various aspects of stress-related behaviors. We hypothesized that the stress response by cocaine- and amphetamine-regulated transcript (CART)-producing EWcp-neurons would depend on the animal’s energy state. To test this hypothesis, we investigated the effects of changes in energy state (mimicked by low, normal and high leptin levels, which were achieved by 24 h fasting, normal chow and leptin injection, respectively) on the response of CART neurons in the EWcp of rats subjected or not to acute restraint stress. Our data show that leptin treatment alone significantly increases CART mRNA expression in the rat EWcp and that in leptin receptor deficient (*db/db*) mice, the number of CART producing neurons in this nucleus is reduced. This suggests that leptin has a stimulatory effect on the production of CART in the EWcp under non-stressed condition. Under stressed condition, however, leptin blunts stress-induced activation of EWcp neurons and decreases their CART mRNA expression. Interestingly, fasting, does not influence the stress-induced activation of EWcp-neurons, and specifically EWcp-CART neurons are not activated. These results suggest that the stress response by the EWcp depends to some degree on the animal’s energy state, a mechanism that may contribute to a better understanding of the complex interplay between obesity and stress.

## INTRODUCTION

In order to maintain homeostasis, vertebrates have to adapt to intrinsic or extrinsic stressors by a highly complicated process in which both neural and endocrine messengers from diverse sources are involved. Depending on the type of stressor, specific stress-sensitive hypothalamic and extrahypothalamic brain centers interact with each other to eventually control the secretion of corticosteroids by the hypothalamic–pituitary–adrenal (HPA)-axis (for references, see e.g., Chrousos and Gold, 1992). These hormones enable the organism to cope with the stress challenge (Sapolsky et al., 2000) but at the same time, urge it to spend a high amount of energy to this adaptation (Kozicz et al., 2011; Morava and Kozicz, 2013). Consequently, the brain needs to be informed about the amount of energy available, so that it can adjust its feeding and metabolic activities and accurately distribute the available energy over essential life processes including adaptation. For this purpose the organism employs various neurochemical brain messengers, such as neuropeptide Y (NPY), insulin, cholecystokinin (CCK), urocortin1 (Ucn1), and nesfatin-1 (e.g., Kalra et al., 1999; Dietrich and Horvath, 2009; Kozicz et al., 2011; Williams and Elmquist, 2012) and ghrelin/leptin-based signaling systems that inform the brain about the amount of peripheral energy information (Zhang et al., 1994; Meier and Gressner, 2004; Roubos et al., 2012). Evidently, prevention and therapy of disorders such as obesity and depression would enormously benefit from a better insight into the ways stress and feeding stimuli are integrated by this complex neuroendocrine signaling system. The present study is concerned with two main players in this system, the anorexigenic peptide, cocaine- and amphetamine-regulated transcript (CART) and the peripheral metabolic hormone, leptin. We focus in particular on the roles of leptin and CART in the stress- and feeding-sensitive extrahypothalamic, centrally projecting Edinger–Westphal nucleus (EWcp).

The EWcp is situated in the rostroventral part of the midbrain, and its activity is strongly influenced by both stressors and the nutritional state that change the neuronal contents of the neuropeptide Ucn1 and Ucn1 mRNA (Gaszner et al., 2004; Kozicz, 2007). The EWcp targets various other stress- and/or feeding-sensitive brain nuclei such as the ventromedial hypothalamus, the lateral septum and the dorsal raphe nucleus, and, moreover, brown adipose tissue (Bittencourt et al., 1999; Ohata et al., 2000; Weitemier et al., 2005; Zhang et al., 2011). Furthermore, lesioning EWcp inhibits food intake (Weitemier and Ryabinin, 2005). Recently, the EWcp has also been demonstrated to receive afferents from different brain regions involved in stress responses and feeding behavior, such as the paraventricular and posterior hypothalamic nuclei and the lateral hypothalamic area (Da Silva et al., 2013). Therefore, the EWcp is supposed to integrate both stress and feeding-related signals in order to contribute to energy-dependent stress adaptation (Kozicz et al., 2011; Xu et al., 2012). In addition to Ucn1, the rodent EWcp produces CART (Kozicz, 2003; Xu et al., 2009), which fully colocalizes with Ucn1 and which mRNA expression is up-regulated by stressors and long-term fasting (Kozicz, 2003; Xu et al., 2009). These data suggest that CART in the EWcp plays a role in integrating stress and feeding signals (Xu et al., 2009).

Involvement of the EWcp in such an integration also appears from the presence of the functional leptin receptor, LepRb (Ahima and Osei, 2004) on 50–60% of the Ucn1 neurons in the rat EWcp (Xu et al., 2011). The 16 kDa adipose derived and blood transported leptin is a product of the *Obese* (*Ob) *gene and an important regulator of energy metabolism; i.e., it reduces food intake and increases energy expenditure (Zhang et al., 1994). The protein acts on LepRb (Ahima and Osei, 2004), which can initiate intracellular signaling cascades (Xu et al., 2011). Leptin can also mediate the stress response, as LepRbs have been identified in stress-sensitive areas (Håkansson and Meister, 1998; Malendowicz et al., 2007). Furthermore, systemic leptin injections improve behavioral impairments in stressed rats (Heiman et al., 1997; Lu et al., 2006). Peripheral leptin administration increases the Ucn1 content of the EWcp, while stimulating STAT3 phosphorylation and inhibiting the electrical activity of these neurons (Xu et al., 2011). The findings above show the complex interplay between leptin and the stress response.

These data together indicate a relationship between stress, leptin signaling and CART expression in the EWcp. The mechanism(s) by which these signals are integrated by the EWcp remain unclear, and with the present study, we aimed to elucidate the link between the EWcp and leptin, fasting and stress. Special attention was placed on the transcriptional and translational dynamics of CART in low (24 h fasting), normal (fed with chow), and high (systemic leptin injection) energy states, and to what extent various energy states would modulate these dynamics under stress conditions. Studies were performed using male rats, wildtype mice and mice lacking the LepRb receptor (*db/db* mice), using semi-quantitative immunocytochemistry and* in situ *hybridization.

## MATERIALS AND METHODS

### ANIMAL HANDLING

Male Wistar-R Amsterdam rats (225–250*g*; bred in the Animal Facility of the Department of Anatomy, Pécs, Hungary) were used for the leptin and stress experiment, and five male C57BL/6J (WT) and five B6.Cg-m+/+Lepr^db^/J (*db/db*) mice (10–12 wk old; obtained from The Jackson Laboratory, Bar Harbor, ME, USA), housed in the Unit for Laboratory Animal Medicine at the University of Michigan, were used for studying the effect of LepRb deficiency. All animals were housed in standard plastic cages, in a temperature- and humidity-controlled environment, on a 12 h light/dark cycle (lights on at 6:00 a.m.) with free access to food and water *ad libitum*. They were allowed 1 week of acclimatization before the start of the experiment. All animal procedures had the approval of the respective University care and use committees.

### PEPTIDE AND ANTISERA

Recombinant mouse leptin was obtained from the National Hormone and Peptide Program (Dr. A. F. Parlow, Los Angeles, CA, USA), mouse anti-CART was a generous gift from Dr. J. T. Claussen (no. Ca6-1 F4D4; Novo Nordisk A/S, Bagsvaerd, Denmark), rabbit anti-c-Fos was from Santa Cruz Biotechnology (no. sc-52, Santa Cruz, CA, USA). Normal donkey serum (NDS), biotinylated donkey-anti-rabbit immunoglobulin (Ig)G and the cyanine^2^ (Cy^2^)-conjugated donkey-anti-mouse, Cy^3^-conjugated donkey-anti-rabbit sera were from Jackson ImmunoResearch (West Grove, PA, USA). ABC Elite solution were purchased from Vector Laboratories (Burlingame, CA, USA). All other immunoreagents were from Sigma Chemical (St. Louis, MO, USA).

### EXPERIMENTAL PROTOCOLS

Experiment 1: kinetics of leptin-induced c-Fos activation, twenty-eight animals were randomly divided into seven equal groups of four animals. Four saline injected rats were sacrificed immediately after intraperitoneal (i.p.) injection (0 h). Other rats were injected i.p. with either leptin (3 mg/kg) or an equal volume saline, and sacrificed 1, 2, 4 h later.

**FIGURE 1:**
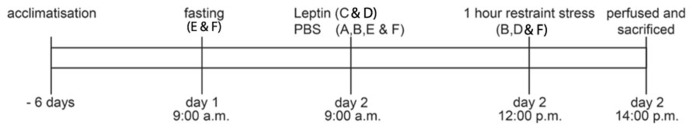
**Timeline showing the animal handling and exposure to the various experimental treatments.** The experiment started on day 1. Letters between brackets indicate the experimental groups (A: PBS + no stress; B: PBS + stress; C: Leptin + no stress; D: Leptin + stress; E: Fasting + no stress; F: Fasting + stress).

Experiment 2: effects of leptin on the stress response of EWcp-CART neurons, thirty rats were divided into six groups based on different treatments (**Figure 1**): PBS injection, leptin injection or fasting, and exposure or no exposure to restraint stress. Rats exposed to a 24 h fasting paradigm (groups E and F) were deprived of rat chow at 9:00 a.m. on day 1 and groups A, B, C, D were fed normally. At 9:00 a.m. on day 2, 3 mg/kg leptin based on previous studies by Münzberg et al. (2003), Huo et al. (2004), Xu et al. (2011) in sterile sodium phosphate-buffered saline (PBS; pH 7.4) was injected i.p. into rats of groups C and D; an equal volume of PBS was injected into controls (groups A and B). To test the effect of the state of energy on the EWcp stress response, rats of groups B, D, and F were subjected to acute restraint stress by placing the animal in a plastic tube (length 200 mm, diameter 45 mm, with several ventilation holes at its side and top) at noon on day 2. Rats not subjected to restraint stress were kept in their home cages.

All the rats were deeply anesthetized with Nembutal (Sanofi, Budapest, Hungary, 100 mg/kg). For experiment 2, after exposing their chest cavity, first a 1 ml blood sample was collected through the left ventricle in an ice-chilled EDTA-containing tube. Next, rats were transcardially perfused with 50 ml 0.1 PBS followed by 250 ml 4% ice-cold paraformaldehyde (PFA) in 0.2 Millonig sodium phosphate buffer (pH 7.4). After decapitation, brains were dissected and stored in PFA fixative, for 2 days. Of each brain, six series of 20 μm thick coronal slices were cut with a Lancer microtome (Ted Pella, Redding, CA, USA) through the entire length of the EWcp (5.0–7.0 mm caudal to Bregma: see Paxinos and Franklin, 2001). Sections were stored in sterile antifreeze solution (0.1 M PBS, 30% ethylene glycol and 20% glycerol) at -20°C. Blood samples were centrifuged at 3000 rpm., for 10 min. A plasma aliquot of 50 μl was stored at -20°C until performing duplicate leptin radioimmunoassay (Linco Research, St. Charles, MI, USA).

Experiment 3: effect of disrupted leptin signaling on CART neurons in the EWcp; five non-stressed WT and five *db/db* mice were deeply anesthetized with i.p. sodium pentobarbital (150 mg/kg), transcardially perfused with ice-cold PBS followed by 4% PFA, for 30 min, decapitated, and brains removed and post-fixed in 4% PFA (Münzberg et al., 2003), for 16 h. Four representative series of coronal sections (30 μm) were cut with a sliding microtome, into a cryoprotective solution (30% ethylene glycol, 30% glycerol; in PBS), and stored at -20°C until use for immunohistochemistry.

### *IN SITU *HYBRIDIZATION

For CART mRNA determination, antisense and sense (control) RNA probes were generated using a full length 520 bp CART cDNA, subcloned in pBluescript (Stratagene, Agilent Technologies, Santa Clara, CA, USA) and labeled with DIG (digoxygenin)-11-UTP using a labeling kit from Roche Molecular Biochemicals (Basel, Switzerland). Sections were fixed in 4% PFA (pH = 7.3) at 4°C for 72 h and rinsed 3 min × 10 min in 0.1 M PBS. Subsequently, the sections were pre-incubated for 10 min at 37°C in proteinase K medium (0.1 M Tris/HCl, 0.05 M EDTA, 0.01 mg/ml proteinase K: Invitrogen, Carlsbad, CA, USA). After rinsing 1 min in autoclaved diethyl pyrocarbonate (DEPC; 100 μl DEPC in 100 ml MQ water) and 1 min in 0.1 M tri-ethanolamine buffer (TEA; pH = 8), acetylation was performed with 0.25% acetic acid anhydride in 0.1 M TEA buffer for 10 min, followed by a 5 min rinse in 2x concentrated standard saline citrate buffer (SSC; pH = 7.0). Hybridization mixture (50% deionized formamide, 0.3 M NaCl, 0.001 M EDTA, Denhardt’s solution, 10% dextran sulfate; pH = 7.0), together with 0.5 mg/ml tRNA and the mRNA-DIG probe (CART: 0.2 ng/ml) were placed in a water bath, at 80°C for 5 min and then on ice for another 5 min. Sections were incubated in hybridization solution, for 16 h at 58°C, rinsed 3 min × 10 min with 4x SSC, incubated with pre-heated RNAse medium (0.5 M NaCl, 0.01 M Tris/HCl, 0.001 M EDTA, 0.01 mg/ml RNAse A: Roche; pH = 8.0) that had been added just before the start of incubation, and rinsed in steps with decreasing SSC concentrations (2x, 1x, 0.5x, 0.1x), for 30 min at 58°C. DIG label was detected with the alkaline phosphatase (AP) procedure with nitroblue tetrazolium chloride/5-bromo-4-chloro-3-indolyl phosphatase-toluidine salt (NBT/BCIP) as substrate. After rinsing 2 min × 10 min in buffer A (0.1 M Tris/HCl, 0.15 M NaCl; pH = 7.5), sections were pre-incubated in Buffer A containing 0.5% blocking agent (Roche) for 1 h, followed by 3 h incubation with sheep anti-DIG-AP (Roche, 1:5.000) in buffer A containing 0.5% blocking agent. Subsequently, sections were rinsed for 2 min × 10 min in buffer A, followed by 2min × 5 min rinsing in buffer B (0.1 M Tris/HCl, 0.15 M NaCl, 0.05 M MgCl_2_; pH = 9.5). After 6 h incubation in NBT/BCIP medium (buffer B, 0.24 mg/ml levamisole: Sigma Chemical, 175 μl NBT/BCIP mixture: Roche) in a light-tight box, the reaction was stopped by washing the sections 2min × 5 min in buffer C (0.1 M Tris/HCl, 0.01 M EDTA; pH = 8.0). Finally, sections were mounted on gelatin-coated glass slides and coverslipped with Kaiser’s glycerol gelatin (Merck, Darmstadt, Germany).

### IMMUNOHISTOCHEMISTRY

We determine relative changes in the amount of substances using semi-quantitative measurements. We used a controlled randomization protocol to make sure that each six-well plate contains sections from one animal per experimental group. In this way, animals belonging to the same group were always assigned in different plates. This procedure minimizes the bias and prevents introducing false positive statistical results. All antibody incubations were performed at the same time using comparable conditions (antibody concentration, incubation time, temperature). Samples from all groups were coded to ensure unbiased data collection. All sections were viewed and confocal settings were determined for the brightest section. All images were collected on the same day using the same settings (for more details, see Image analysis). Diaminobenzidine (DAB) immunohistochemistry was performed in Experiment 1 and fluorescent immunohistochemistry was performed in Experiment 2 and 3.

For c-Fos immunuhistochemistry with DAB, sections were washed 4 min × 15 min in 0.1 M PBS followed by 0.5% Triton X-100 in PBS for 30 min to enhance antigen penetration. After an additional 15 min wash in PBS, sections were incubated in 1% H_2_O_2_ for 10 min. After 3 min × 5 min washes in PBS, the sections were placed for 1 h into a solution of 2% NDS to block non-specific binding sites. After a brief wash in PBS, the sections were transferred into vials containing the primary polyclonal (rabbit) anti c-Fos antibody at a dilution of 1:2000 overnight. The next day, after 4 min × 15 min washes in PBS, sections were incubated into biotinylated donkey-anti-rabbit immunoglobulin (Ig)G (1:200) for 1 h and subsequently into ABC Vector (1:200) for 1 h. The c-Fos signal was visualized with adding 10 mg DAB and 35 mg ammonium-nickel-sulfate in 50 ml Tris buffer (pH 7.6). The reaction was stopped after 9 min in Tris buffer. The sections were washed and mounted on gelatin coated slides and air dried. After drying they were dehydrated by gradual steps of alcohol, iso-propanol and xylene and mounted with Entallan.

For single immunolabeling of CART, sections were treated with 0.5% Triton X-100 in PBS, for 30 min, blocked in 2% NDS for 1 h, and incubated in primary monoclonal mouse anti-CART (1:1500) overnight. This was followed by 2 h incubation with secondary Cy^2^-conjugated anti-mouse IgG (1:100). Finally, sections were rinsed 3 min × 10 min in PBS, mounted on gelatin-coated glass slides, air-dried and coverslipped with FluorSave reagent (EMD Biosciences, San Diego, CA, USA).

For double-immunolabeling of CART and c-Fos, sections were processed as described for single immunofluorescent labeling but with incubation in a mixture of primary monoclonal mouse anti-CART (1:1500) and polyclonal rabbit anti-c-Fos serum (1:800) overnight and then in a mixture of Cy^3^-conjugated donkey anti-rabbit IgG antiserum (1:100) and Cy^2^-conjugated donkey anti-mouse IgG antiserum (1:100) in 2% NDS for 2 h.

The high specificities of the mouse anti-CART (Koylu et al., 1997) and rabbit anti-c-Fos (Gaszner et al., 2004; Korosi et al., 2005) sera have been previously reported.

### IMAGE ANALYSIS

Immunostainings were studied with a DM IRE2 inverted epifluorescence microscope (Leica Microsystems, Mannheim, Germany) attached to a TCS SP2-AOBS confocal laser scanning unit (Leica Microsystems, Wetzlar, Germany) using a 488 nm Argon laser, a 561 nm orange laser and a x20 dry objective. The fluorescent signal from each image was thresholded at the same level to eliminate saturation. For double immunofluorescence measurements, Images were taken using sequential scanning for each channel, with the same settings in laser intensity, detector gain and amplifier offset. Images were saved in tagged image file format (TIFF) to prevent loss of information. For semi-quantitative determination of the amounts of CART and c-Fos protein contents in the EWcp, two parameters were determined using Image J software version 1.41 (NIH, Bethesda, MD, USA): (1) the representative number of immunoreactive neurons per section generated by averaging three sections of the midlevel of the EWcp (bregma -5.5 to -6.4 mm; Paxinos and Franklin, 2001), and (2) per neuron, the specific immunoreactivity signal density (SSD) averaged over all neurons present in the sections. The SSD was corrected for background density outside the EWcp, and expressed in arbitrary units (AUs) per neuron.

### STATISTICAL ANALYSIS

Data are presented as the mean ± standard error of the mean (SEM). To compare different conditions, two-way analysis of variance (ANOVA with independent variables “leptin” and “stress”) with Bonferroni post-test was performed using Graphpad Prism version 5.04 for Windows (GraphPad Software, La Jolla, CA, USA), after appropriate transformation of some data on the basis of Levene’s test for homogeneity of variance (Levene, 1960). For comparison of WT with *db/db* mice, student’s *t*-test was performed. In all cases, *p* < 5% was considered to be significant.

## RESULTS

### EXPERIMENT 1: KINETICS OF LEPTIN-INDUCED c-Fos ACTIVATION IN EWcp

**FIGURE 2:**
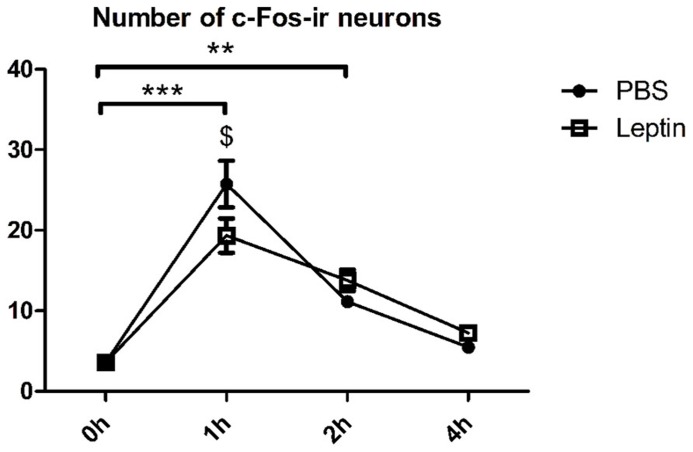
**The number of c-Fos-ir neurons in the EWcp after administration of PBS or leptin for 0, 1, 2, and 4 h.** Vertical bars represent the means ± SEM; *N* = 4. Significant difference between groups treated for different periods with PBS and leptin is marked by “*”; significant difference between leptin and PBS group is marked by “$”. $*P* < 0.05; **P < 0.01; ***P < 0.005.

**Figure 2** shows the time effect of injected leptin on the activity of EWcp, as measured by c-Fos immunoreactivity (i.r.). Two-way ANOVA revealed significant effects of time (*F*_3,24_ = 70.7; *P* < 0.0001) and time × leptin interaction (*F*_3,24_ = 4.2; *P* < 0.05). *Post hoc *test revealed that at 1 h after injection, the EWcp exhibited increased number of c-Fos-ir neurons. This increase was significantly higher in the PBS injected animals compared with leptin injected ones (PBS: 7.2 times; leptin: 5.4 times). We observed fewer c-Fos-ir neurons 2 h after injection (either PBS or leptin) vs. 1 h post-injection, however, more c-Fos-ir neurons when compared with baseline (PBS: 3.1 times; leptin: 3.8 times). Four hours after injection of either PBS or leptin, the number of c-Fos-ir neurons was not different between 0 h and 4 h post-injection.

### EXPERIMENT 2: LEPTIN’S EFFECT ON STRESS-INDUCED ACTIVATION OF EWcp-CART NEURONS

#### Leptin plasma measurement

**FIGURE 3:**
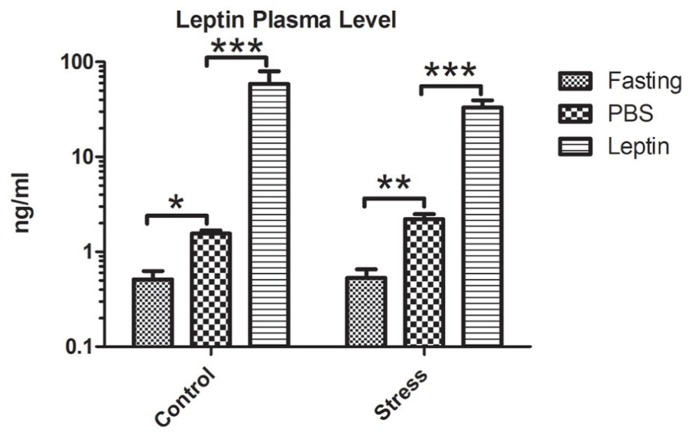
**Leptin plasma level in the various experimental groups.** Vertical bars represent the means + SEM; *N* = 5. Asterisks indicate significant differences between fasting, PBS and leptin treated groups. *P < 0.05; **P < 0.01; ***P < 0.005.

The leptin plasma concentrations 5 h after injecting either PBS or leptin are presented in **Figure 3**. ANOVA showed a main effect of leptin injection (*F*_2,24_ = 79.13; *P* < 0.0001). *Post hoc* analysis revealed that in both non-stressed and stressed rats, plasma leptin was significantly lower after fasting (*P* < 0.05) and higher after leptin-injection (*P* < 0.001) compared with PBS-injected controls.

#### c-Fos immunofluorescence and activation of CART expressing neurons

**FIGURE 4:**
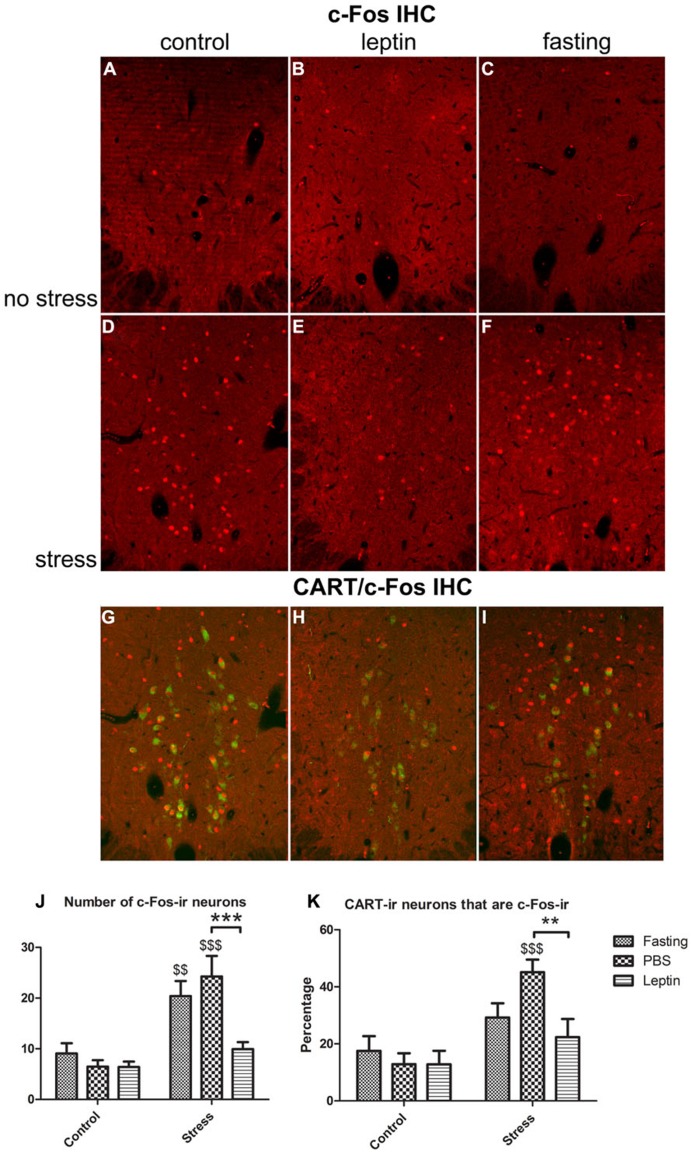
**Activation of EWcp CART expressing neurons by restraint stress.** **(A–C)** c-Fos-ir in fasting, PBS-injected and leptin-injected rats, **(D–F)** the same three treatments, respectively, but stressed. **(G–I)** Merged images of fluorescent double labeling showing CART- and c-Fos-ir in EWcp neurons. **(J)** Quantitative analysis of stress-induced activation of EWcp neurons and **(K)** percentage of CART neurons exhibiting c-Fos-ir in the various experimental groups. Vertical bars represent the means + SEM; *N* = 5. Asterisks with lines indicate significant differences between fasting, PBS and leptin treated groups. **P < 0.01; ***P < 0.005; dollar signs alone indicate significant differences of stressed group with respective control group. $$*P* < 0.01; $$$*P* < 0.005. Scale bars: 50 μm.

The general activation of the EWcp in response to a changed peripheral energy level and/or stress was determined by counting the number of c-Fos-ir neurons (**Figures 4A–F**). ANOVA showed main effects of leptin (*F*_2,24_ = 5.3, *P* < 0.05), stress (*F*_1,24_ = 35.83, *P* < 0.0001) and leptin × stress interaction (*F*_2,24_ = 3.89, *P* < 0.05; **Figure 4J**). Although the number of c-Fos-ir neurons was not different between fasted, PBS- or leptin-treated animals under non-stressed condition, it was noticeably higher in stressed rats in either fasted (*P* < 0.01) or PBS-treated (*P* < 0.001) animals. It is noteworthy that leptin injection significantly blunted stress-induced neuronal activation. More specifically, c-Fos-ir number was 2.5 times higher in fasted, 3.75 higher in PBS injected and only 1.55 higher in leptin injected rats compared with control (no stress) condition.

In order to test for activation of CART-neurons, we determined the percentage of CART-containing neurons that also exhibited c-Fos-ir (**Figures 4G–I**). We found significant effects of stress (*F*_1,24_ = 19.19, *P* < 0.005) and leptin × stress interaction (*F*_2,24_ = 3.2, *P* < 0.05; **Figure 4K**). *Post hoc *analysis revealed that stressed PBS-treated rats had approximately 3.5 times more c-Fos-ir in EWcp CART-ir neurons vs. non-stressed PBS-treated rats (*P* < 0.001); whereas leptin treatment and fasting significantly blunted stress-induced activation of EWcp CART-ir neurons (**Figure 4K**).

#### Quantification of CART mRNA and peptide amounts

**FIGURE 5:**
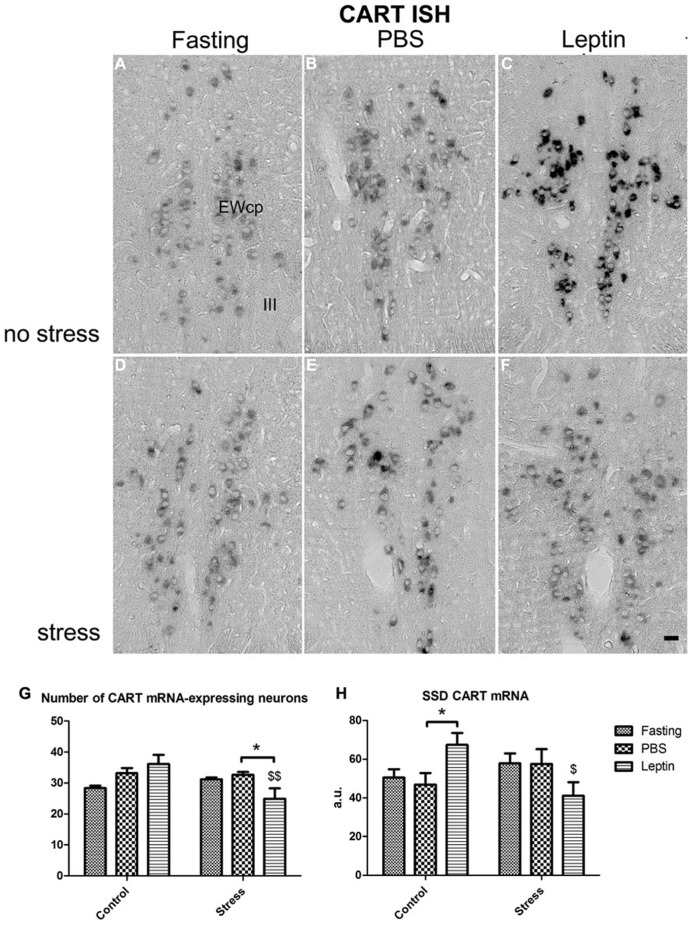
***In situ* hybridization of CART mRNA in EWcp neurons. (A–C)** Fasting, PBS-injected and leptin-injected rats, **(D–F)** the same three treatments, respectively, but stressed. **(G)** Number of CART mRNA expressing neurons and **(H)** SSD per perikaryon, expressed in arbitrary units (a.u.). Vertical bars represent the means + SEM; *N* = 5. Asterisks with lines indicate significant differences between fasting, PBS and leptin treated groups. *P < 0.05; dollar signs alone indicate significant differences of stressed group with respective control group. $*P* < 0.05; $$*P* < 0.001. Scale bars: 50 μm.

To test if leptin, fasting or stress have an effect on the transcriptional activity of CART in the EWcp, we performed *in situ *hybridization(**Figures 5A–H**). After counting the number of mRNA-expressing neurons and measuring the hybridization signal density (SSD), ANOVA (**Figures 5G,H**) revealed a main effect of leptin × stress interaction (*F*_2,21_ = 5.81; *P* < 0.01; *F*_2,21_ = 5.63; *P* < 0.05 respectively).* Post hoc *analysis showed that in the non-stressed condition, leptin treatment increased the SSD of CART mRNA (*P* < 0.05), but in the stressed condition, the same treatment decreased the number of CART mRNA-expressing neurons (*P* < 0.05). When comparing stressed with non-stressed rats, injection of leptin significantly lowered the number and SSD of CART mRNA-positive neurons (*P* < 0.01 and *P* < 0.05, respectively).

**FIGURE 6:**
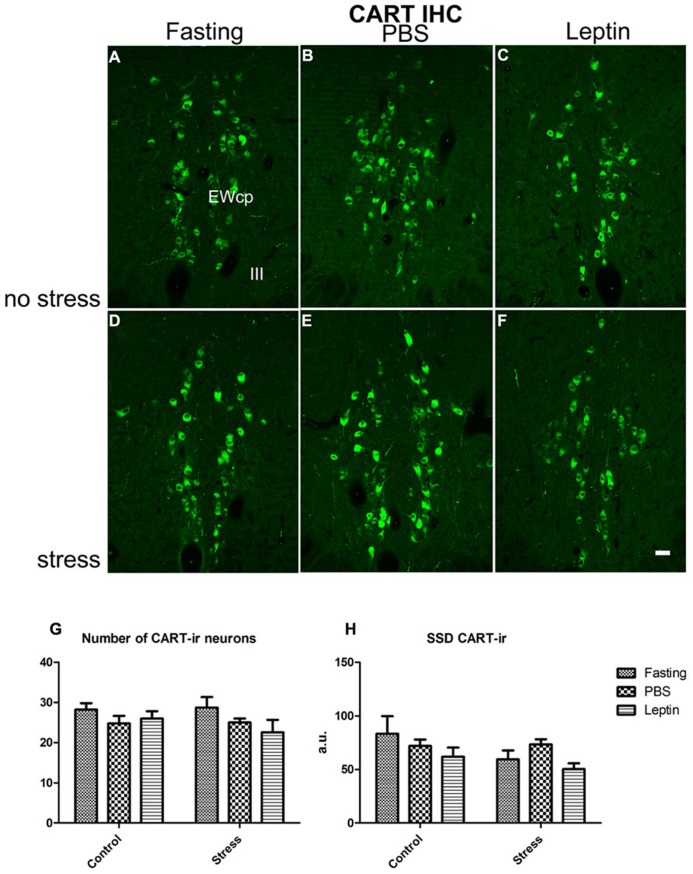
**Immunofluorescence labeling of EWcp neurons expressing CART.** **(A–C)** CART-ir in fasting, PBS-injected and leptin-injected rats, **(D–F)** the same three treatments, respectively, but stressed. **(G)** Number of CART-ir and **(H)** SSD per perikaryon, expressed in arbitrary units (a.u.). Vertical bars represent the means + SEM; *N* = 5. No significant differences were present. Scale bars: 50 μm.

Next, we assessed the amount of CART-ir neurons in the EWcp using semi-quantitative immunohistochemistry (**Figures 6A–F**). We counted the number of immunopositive perikarya as well as measured SSD per perikaryon (**Figures 6G,H**) to estimate CART peptide content. We found no difference for any of these parameters.

### EXPERIMENT 3: EFFECTS OF DISRUPTED LEPTIN SIGNALING ON EWcp-CART NEURONS

**FIGURE 7:**
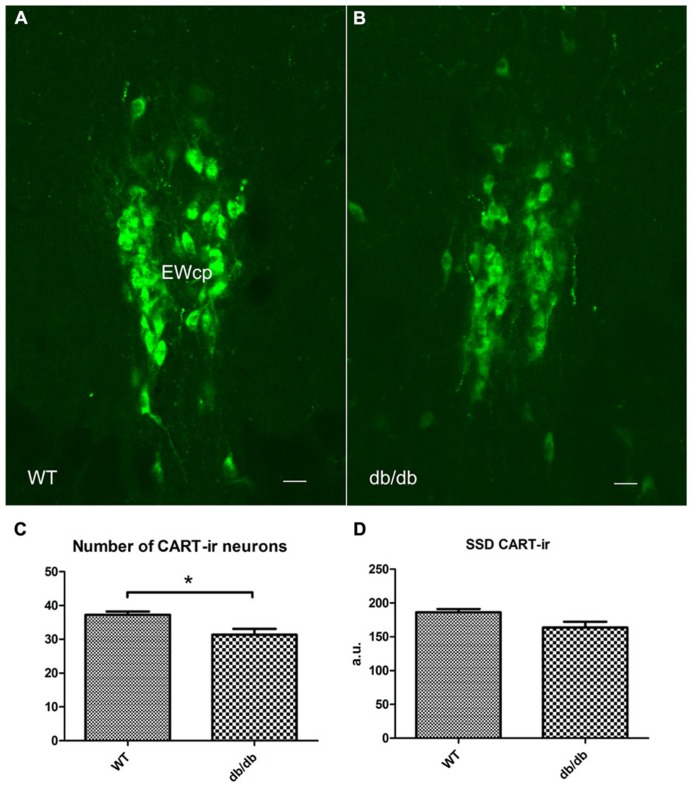
**CART content in the EWcp by disrupted leptin signaling.** **(A,B)**, fluorescent immunohistochemistry shows CART-ir in the EWcp in WT and *db/db* mice. **(C,D)**, the number and SSD of CART-ir neurons are decreased in *db/db *mice compared with WT. Scale bars: 20 μm. *P < 0.05.

Finally, to assess the effect of leptin signaling deficiency, we compared CART-ir immunoreactivity in the EWcp of *db/db* mice with that of wild type littermates (WT; **Figures 7A,B**). We observed a lower number of EWcp-CART-ir neurons in *db/db* mice (*P* < 0.05; **Figure 7C**) and a strong tendency toward lower SSD of CART-ir in *db/db* mice (*P* = 0.05; **Figure 7D**).

## DISCUSSION

Based on the expression of LepRb in the EWcp and the involvement of EWcp in stress response and energy balance, we hypothesized that EWcp neurons would respond to stress differentially under various energy states mimicked by low, normal and high plasma leptin levels. The present study demonstrates that leptin not only attenuates the overall activation of EWcp neurons, but it also inhibits the activation of EWcp-CART neurons in response to acute (restraint) stress. Interestingly, although fasted rats and normal fed animals exhibited comparable activation pattern of EWcp neurons, in fasted animals this activation did not include EWcp-CART neurons.

As we aimed to investigate the interaction of leptin and stress in the EWcp, we needed to minimize the effect of the initial injection stress and maximize the effect of leptin. For this reason, we have assessed the kinetics of c-Fos expression in the EWcp after leptin injection. The activation of c-Fos by PBS or leptin injection within 1 h most probably represents an acute stress response. Interestingly, this initial c-Fos response was dampened by leptin, an effect that was transient and disappeared within 2 h. Whether the dampening effect of leptin on stress-associated activation of c-Fos in the first hour is due to a direct inhibitory action, remains to be investigated. At 4 h, in both PBS and leptin injected animals, c-Fos was not activated anymore in the EWcp. Therefore, we conclude that leptin alone does not result in c-Fos activation in the EWcp. Our previous study showed that pSTAT3 activation reaches its peak in the EWcp 2 h after leptin injection (Xu et al., 2011). Based on these data, we decided to subject the rats to restraint stress 3 h after leptin injection in Experiment 2.

In Experiment 2, we have assessed the interaction of leptin and stress in the EWcp. Under non-stressed, basal conditions, systemic leptin injection does not change the general activity of the EWcp. However, leptin injection did up-regulate CART mRNA production in the EWcp. These results suggest that leptin has a specific stimulatory effect on the production of CART in the EWcp. This notion is corroborated by the present data obtained with *db/db* mice, which lack the LepRb and show a lower CART content in the EWcp than WT mice. It is well established that leptin, by acting on receptors in various parts of the brain, reduces food intake, thereby causing body weight loss (e.g., Campfield et al., 1995; Gorska et al., 2010). In the EWcp, leptin acts on LepRb in CART/Ucn1 neurons and activates the JAK2-STAT3 pathway (Xu et al., 2011). In addition, the presence of a STAT-binding motif in the CART gene promoter suggests that this gene could be regulated directly via cytokine signaling (Dominguez et al., 2002). These, taken together with the fact that CART exerts an anorexigenic effect (Rogge et al., 2008), the stimulatory action of leptin on CART mRNA expression would account for leptin’s inhibitory effect on food intake.

Restraint stress appears to activate about 50% of the EWcp CART neurons. However, this activation is not accompanied by the induction of CART gene. One might suggest that the induction of CART mRNA by stress needs longer time to occur. This is possible, however, not very likely, because previous studies have shown that 2 h after initiation of various acute stressors (e.g., pain, restraint or foot shock), Ucn1 mRNA in the EWcp can be significantly up-regulated, accompanied by increased expression of c-Fos (Kozicz et al., 2001; Cespedes et al., 2010; Rouwette et al., 2011). Therefore, we suggest that CART mRNA, in contrast to that of Ucn1, is not induced by acute restraint stress, a hypothesis that needs further investigation.

In the present study, we found that leptin injection strongly attenuates restraint stress-induced activation of EWcp neurons, which occurs concomitantly with attenuated CART mRNA expression. This could represent an important mechanism by which leptin participates in the regulation of stress response. Leptin has been reported to produce antidepressant- (Lu et al., 2006; Lu, 2007) and anxiolytic-like (Liu et al., 2010) effects in rats and mice. However, these behavioral studies were performed in either non-stressed or chronically stressed animals. So far, only one study has addressed the effect of leptin on acute stress-induced behavioral deficits (Haque et al., 2013). This study has shown that immobilization stress-induced anorexia and decrease in body weight can be reversed by leptin injection (Haque et al., 2013). These results might be striking at the first sight and are not explainable in terms of the conventional function of leptin (i.e., reducing food intake). However, this inhibitory action of leptin on stress-induced anorexia could well represent an anxiolytic/antidepressant-like effect. Here, we demonstrate that leptin blunts the activation of EWcp neurons and decreases CART mRNA expression in stressed rats. This may indicate that CART is a downstream component of a leptin-regulated mechanism that reduces anxiety-related behavior under stress conditions.

The role of midbrain CART in stress is further corroborated by the fact that CART in the rat EWcp was significantly elevated after applying a two-week mild stress paradigm (Xu et al., 2010). In two different rat models for depression, it was noted that depressive-like behavior correlated with a drastic reduction in CART-immunoreactivity not only in the hypothalamic paraventricular and arcuate nuclei but also in the EWcp. Moreover, CART treatment could reverse depression-like phenotypes (Dandekar et al., 2009; Wiehager et al., 2009). The association between CART and mood disorders has also been suggested. Specifically, CART mRNA expression was markedly higher in the EWcp in depressed suicide victims vs. controls (Bloem et al., 2012). Although these results do not permit to conclude whether CART in the EWcp is anxiogenic or anxiolytic, they collectively position midbrain CART as a possible modulator of stress-related behavior.

Another notable observation is that leptin down-regulates CART mRNA in stressed condition, but not CART peptide contents. We found similar CART peptide and mRNA dynamics in mouse exposed to acute restraint stress (Okere et al., 2010). The dissociation between CART mRNA and CART peptide content may be explained by assuming that leptin not only inhibits CART mRNA expression, but it attenuates too the axonal transport of CART peptide out of the cell body. This would leave the amount of CART peptide stored in the cell body unchanged.

In contrast to the strong attenuating effect of leptin on stress-induced activation of EWcp, the activation of EWcp by stress was comparable between fasted and normal fed rats. Interestingly, when we assessed the phenotype of neurons recruited by stress, CART neurons were strongly activated by stress in normal fed rats, but remained inactive in fasted rats. This suggests that another population of EWcp neurons is activated upon stress under fasted conditions. In the absence of food, another fuel signal, ghrelin, is released from the stomach to strongly stimulate food intake (Date et al., 2000; Tschöp et al., 2000). Ghrelin receptor protein as well as its mRNA are abundantly present in the rat EWcp (Zigman et al., 2006; Spencer et al., 2012). Taken together, it is plausible that ghrelin, induced by 24 h fasting, would specifically induce stress-associated activation of non-CART neurons in the EWcp, and/or inhibit the activation of CART neurons in EWcp.

In conclusion, here we show that the EWcp CART neurons respond differently to acute stress under fasted, normally sated (normal chow diet) and highly sated (artificially mimicked by i.p. leptin injection) conditions. We suggest that this mechanism may play a physiological role in the central integration of stressful and peripheral fuel signals. Such a mechanism would allow an animal to prepare the appropriate stress response under various energy states. Consequently, failure of this mechanism could contribute to the pathogenesis of feeding-related and stress-induced disorders.

## Conflict of Interest Statement

The authors declare that the research was conducted in the absence of any commercial or financial relationships that could be construed as a potential conflict of interest.
